# Microarray Identifies a Key Carcinogenic Circular RNA 0008594 That Is Related to Non-Small-Cell Lung Cancer Development and Lymph Node Metastasis and Promotes NSCLC Progression by Regulating the miR-760-Mediated PI3K/AKT and MEK/ERK Pathways

**DOI:** 10.3389/fonc.2021.757541

**Published:** 2021-11-11

**Authors:** Qiushi Wang, Chunhua Yan, Pengfei Zhang, Guanghua Li, Ruidong Zhu, Hanbing Wang, Libo Wu, Guangquan Xu

**Affiliations:** ^1^ The Second Department of General Thoracic Surgery, The Second Affiliated Hospital of Harbin Medical University, Harbin, China; ^2^ Department of Respiratory, Longgang District People’s Hospital of Shenzhen, Shenzhen, China; ^3^ Department of Respiratory, Longgang District The Third People’s Hospital of Shenzhen, Shenzhen, China

**Keywords:** circular RNA, microarray, non-small-cell lung cancer, circ_0008594, miR-760

## Abstract

**Purpose:**

This study aimed to explore the circular RNA (circRNA/circ) profile engaged in non-small cell lung cancer (NSCLC) development and metastasis and to investigate potentially key carcinogenic circRNAs related to NSCLC.

**Methods:**

CircRNA profiles between 10 NSCLC tissues and 10 adjacent tissues and between five NSCLC tissues with lymph node metastasis (LNM) and five NSCLC tissues without LNM were detected by Arraystar Human circRNA Array followed by bioinformatics. Circ_0008594 knockdown, circ_0004293 overexpression, and circ_0003832 overexpression plasmids were transfected into H23 and H460 cells to sort potential oncogenic circRNA. Then circ_0008594 overexpression and knockdown plasmids were transfected, followed by that circ_0008594 knockdown plus miR-760 knockdown plasmids were transfected into these cells. Cell proliferation, apoptosis, invasion, stemness, and pathways were detected. In addition, xenograft mice models were constructed *via* injecting H23 cells with circ_0008594 overexpression or knockdown to validate the findings.

**Results:**

A total of 455 dysregulated circRNAs in NSCLC tissues *versus* adjacent tissues and 353 dysregulated circRNAs in NSCLC tissues with LNM *versus* those without LNM were discovered. *Via* cross-analysis, 19 accordant circRNAs were uncovered, among which three candidate circRNAs (circ_0008594, circ_0004293, circ_0003832) were chosen for functional experiments, during which it was observed that circ_0008549 affected H23 and H460 cell proliferation and apoptosis more obviously than circ_0004293 and circ_0003832. Subsequent experiments showed that circ_0008594 promoted H23 and H460 cell proliferation and invasion but affected stemness less and negatively regulated miR-760 *via* direct binding. Furthermore, miR-760 attenuated the effect of circ_0008549 on regulating H23 and H460 cell functions and the PI3K/AKT and MEK/ERK pathways. *In vivo* experiments further confirmed that circ_0008549 increased tumor volume, epithelial-mesenchymal transition, and the PI3K/AKT and MEK/ERK pathways while reducing tumor apoptosis and miR-760 NSCLC xenograft models.

**Conclusion:**

Our study identifies several valuable circRNAs related to NSCLC development and LNM. Furthermore, as a key functional circRNA, circ_0008594 was observed to promote NSCLC progression by regulating the miR-760-mediated PI3K/AKT and MEK/ERK pathways.

## Introduction

Lung cancer remains the deadliest and second most frequent cancer worldwide, accounting for 11.4% of newly diagnosed cancer cases and 18.0% of cancer-related deaths in 2020 (1). As the most common type of lung cancer, non-small-cell lung cancer (NSCLC) accounts for approximately 85% of all cases (2, 3). NSCLC treatment has been greatly promoted in recent years, benefiting from measures such as early screening programs, treatment strategy improvement, individualized and precise medicine, and novel targeted drug development (4–7). In particular, along with the progression of molecular biotechnology, emerging treatment targets have been identified, and corresponding inhibitors have been developed, such as epidermal growth factor receptor (EGFR) tyrosine kinase inhibitors (TKIs), anaplastic lymphoma kinase (ALK) TKIs, and antiangiogenic drugs (8–10). However, even though the above improvements have been realized, the prognosis of NSCLC is still dismal; therefore, efforts continue to explore the underlying pathogenesis of NSCLC to develop more treatment targets to further prolong the survival of NSCLC.

Circular RNA (circRNA/circ), a recently identified non-coding RNA with a loop construction, regulates numerous cell functions and biological processes and participates in the pathogenesis of almost all cancers (11–15). In terms of NSCLC, several specific functional circRNAs have been discovered to regulate NSCLC growth, metastasis, and drug sensitivity *via* various oncogenes and carcinogenic pathways (16–19). However, there remain a large number of circRNAs whose functions or involvement in NSCLC need to be evaluated, and only a very limited number of studies have explored the comprehensive circRNA profile engaged in NSCLC etiology (20, 21).

Therefore, the current study assessed the dysregulated circRNA profile between NSCLC tissues and adjacent non-cancerous tissues and between NSCLC tissues with lymph node metastasis (LNM) and those without LNM to identify candidate circRNAs related to both NSCLC development and LNM. Subsequently, *via* functional experiments, a key carcinogenic circRNA, circ_0008594, was identified, and its effect on NSCLC growth, invasion, and stemness as well as its interaction with microRNA (miRNA/miR)-760 and the PI3K/AKT and MEK/ERK pathways were investigated *in vitro* and *in vivo.*

## Methods

### CircRNA Array Analysis

Tumor and adjacent tissue samples from five NSCLC patients with LNM and five NSCLC patients with non-LNM (NLNM) were collected after approval by the Ethics Committee with approval No. KY2018-244. Arraystar Human circRNA Array chip (Agilent, USA) analysis was performed by Genergy Bio (Shanghai, China). The sample preparation and microarray hybridization were detected according to Arraystar’s protocols. Quantile normalization, data processing, bioinformatics analysis, and graph plotting were performed using R (Version 3.6.3). Briefly, principal component analysis (PCA) was performed using the “factoextra” package. The “limma” package was used to analyze differentially expressed circRNAs (DECirc). CircRNAs with a fold change (FC) >2.0 and adjusted *P* value <0.05 were considered DECircs. Heatmap plots were analyzed using the “Pheatmap” package. Enrichment of Gene Ontology (GO) terms and Kyoto Encyclopedia of Genes and Genomes (KEGG) pathways in DECircs based on located genes were performed by Fisher’s exact test. Accordant circRNAs in the comparison of tumor *vs.* adjacent and LNM *vs.* NLNM were ranked by the absolute mean value of Log_2_FC and exhibited by Venn plots. The circRNA-miRNA co-network was constructed based on the miRanda database according to a previous study (22).

### Cell Lines and Culture Conditions

NSCLC cell lines (A549, H23, H460, H522, and H1299), a normal human lung bronchial epithelial cell line (BEAS-2B), and a 293T cell line were obtained from the American Type Culture Collection (ATCC). H23, H460, H522, and H1299 cells were cultured in RPMI1640 (Sigam, USA). A549, BEAS-2B, and 293T cells were cultured in DMEM (Sigam, USA). All media were supplemented with 10% fetal bovine serum (Gibco, USA), and all cells were maintained at 37°C under 5% CO_2_.

### Plasmids and Antibodies

The circ_0008594, circ_0004293, circ_0003832, miR-760 interference or overexpression plasmids, and negative control (NC) plasmids were obtained from GenePharma (Shanghai, China). Anti-PI3K, anti-p-PI3K, anti-AKT, and anti-p-AKT antibodies were purchased from Abcam (Cambridge, USA). Anti-MEK1/2, anti-p-MEK1/2, anti-ERK1/2, anti-p-ERK1/2, anti-E-cadherin, anti-vimentin, and anti-Snail antibodies were purchased from Cell Signaling Technology (Boston, USA). Anti-GAPDH and secondary antibodies were purchased from Affinity (Changzhou, China). The antibody information is listed in **Supplementary Table 1**.

### CircRNA Validation Experiment

BEAS-2B, A549, H23, H460, H522, and H1299 cells were cultured and harvested for quantitative reverse transcription polymerase chain reaction (qRT–PCR) assay to validate the circRNA expression. The circ_0008594, circ_0004293, circ_0003832, and NC interference or overexpression plasmids were transfected into H23 and H460 cells using Lipofectamine^®^ 3000 (Invitrogen, USA) according to the manufacturer’s instructions. At 48 hours (h) after transfection, cells were harvested for qRT–PCR and apoptosis assays. Cell proliferation assays were performed at 0, 24, 48, and 72 h after transfection.

### Circ_0008594 Regulation Experiment

The cultured H23 and H460 cells were divided into five groups: a Normal group (without any transfection); an NC(+) group (transfected with NC overexpression plasmid); a Circ(+) group (transfected with circ_0008594 overexpression plasmid); an NC(−) group (transfected with NC interference plasmid); and a Circ(−) group (transfected with circ_0008594 interference plasmid), respectively. qRT–PCR, cell proliferation, apoptosis, invasion, and sphere formation assays were performed after transfection.

### Luciferase Reporter Assay

Circ_0008594 wild-type (WT) and mutant (Mut) plasmids were constructed using the pGL6 plasmid (GenePharma, China). The 293T cells were seeded in 12-well plates (2 × 10^5^ cells/well). The circ_0008594 WT or circ_0008594 Mut plasmids (50 ng/well) were co-transfected with miR-760 or NC mimics (20 nM) (GenePharma, China) into 293T cells using Lipofectamine^®^ 3000. Then cells were assayed with Luciferase Assay kit (Promega, USA) after 48 h culture according to the manufacturer’s instruction. Luciferase activity was normalized to Renilla luciferase activity.

### Rescue Experiment

H23 and H460 cells were cultured and divided into five groups: a Normal group (without any treatment); an NC(−) group (transfected with NC interference plasmid); a Circ(−) group (transfected with circ_0008594 interference plasmid); an miR(−) group (transfected with miR-760 interference plasmid); and a Circ(−) and miR(−) group (transfected with circ_0008594 and miR-760 interference plasmid), respectively. Cells were harvested for qRT–PCR, proliferation, apoptosis, invasion, sphere formation, and western blot assays.

### Drug Treatment

To assess whether circ_0008594 regulated tumor development *via* PI3K/AKT and MEK/ERK pathways, the cells were transfected with circ_0008594 overexpression plasmid and cultured with 1 μM ipatasertib (Sigma, USA) or 30 nM trametinib (Sigma, USA). Briefly, H23 and H460 cells were cultured and divided into four groups, namely, NC(+) group (transfected with NC overexpression plasmid), Circ(+) group (transfected with circ_0008594 overexpression plasmid), Circ(+) and Ipatasertib group (transfected with circ_0008594 overexpression plasmid and treated with ipatasertib), and Circ(+) and Trematinib group (transfected with circ_0008594 overexpression plasmid and treated with trametinib). Cells were harvested for proliferation, apoptosis, and invasion assays.

### Cell Proliferation, Apoptosis, and Invasion Assays

Cell proliferation assays were carried out at 0, 24, 48, and 72 h after transfection using a Cell Counting Kit-8 (Dojindo, Japan). Briefly, 4×10^3^ cells were seeded on a 96-well plate. After transfection, cells were incubated with 10 μl of reagent for 2 h. The absorbance was measured at wavelengths of 450 nm. A TUNEL cell apoptosis kit (Beyotime, China) was used to analyze cell apoptosis after transfection. Briefly, the cells were fixed with 4% paraformaldehyde, permeabilized with a Triton X-100 kit (Beyotime, China), and incubated with TUNEL solution for 0.5 h. Fluorescence was observed and analyzed using a fluorescence microscope (OLYMPUS, Japan). Transwell assays were used to assess cell invasion ability. Briefly, the Transwell insert (Corning, China) was precoated with Matrigel (Corning, China). Then, 4×10^4^ cells were seeded into Transwell inserts and cultured for 24 h. Afterward, the invasive cells were fixed with formaldehyde (Beyotime, China) and stained with crystal violet (Sangon, China).

### Sphere Formation Assay

A total of 1×10^3^ cells were seeded into six-well ultralow attachment dishes (Corning, USA) in DMEM/F12 (Sigma, USA) containing 20 ng/ml EGF (Gibco, USA), 20 ng/ml bFGF (Gibco, USA), and 2% B27 (Gibco, USA). Cells were incubated for 12 days, and the numbers of spheres (diameter >50 μm) were counted under a microscope (Olympus, Japan).

### Western Blot

Cell lysis buffer (CST, USA) was utilized for total protein extraction of cells. Total protein was quantified using a BCA Kit (Bio–Rad, USA) and was then separated using a 4–20% polyacrylamide gel (Willget, China). After the proteins were transferred to membranes for 1.5 h, the membranes were blocked for 1 h and then incubated with primary antibodies and secondary antibodies successively. The bands were luminesced using ECL solution (Vazyme, China) and exposed to X-ray film (Carestream, Canada).

### Xenograft Nude Mouse Model

Twenty-four 6-week-old BALB/c nude mice were obtained from SLAC Co., Ltd. (Shanghai, China) and randomly divided into four groups (n=6 for each group): a Normal group (injected with normal H23 cells); an NC group (injected with H23 cells transfected with lentivirus control); a Circ(+) group (injected with H23 cells that overexpressed circ_0008594); and a Circ(−) group (injected with H23 cells that knocked down circ_0008594). Briefly, H23 cells that stably overexpressed or knocked down circ_0008594 and were transfected with lentivirus control were resuspended in PBS (100 μl; 4×10^7^ cells/ml). Then, cells were subcutaneously injected into the dorsal thighs of the nude mice. Tumor size was measured every 3 days and calculated using the formula V = 0.5 × (longer tumor diameters) × (shorter tumor diameters) ^2^. Nude mice were euthanized after 30 days. Tumor samples were collected for qRT–PCR, TUNEL, and immunohistochemical (IHC) analyses. The animal experiments were approved by the Animal Care Committee with approval No. HMDW173 and conducted with the guidelines of the Care and Use of Laboratory Animals.

### qRT–PCR

Total RNA in cells and tumor samples was extracted with TRIzol (Invitrogen, USA). For the transcription of circRNAs, the linear RNA was pre-removed by RNase (Epicentre, USA). Linear RNA removal was not conducted for the transcription of miRNA. Transcription and qPCR were completed with the QuantiNova Reverse Transcription Kit (Qiagen, Germany) and qPCR Mix (Takara, Japan). The results were calculated using the 2^−ΔΔCt^ method with U6 and GAPDH serving as internal references for miRNA and circRNA. The primers are listed in **Supplementary Table 2**.

### TUNEL and IHC Staining

The tumor samples were fixed with 4% paraformaldehyde, embedded in paraffin, and sliced into 4-μm sections. A TUNEL Apoptosis Assay Kit (Beyotime, China) was applied for apoptosis rate analyses following the manufacturer’s instructions. For IHC staining, the sections were incubated with primary and secondary antibodies consecutively. After DAB staining, the sections were observed under a microscope (Olympus, Japan). The IHC score was calculated according to a previous study (23).

### Statistical Analysis

GraphPad Prism 7.0 (GraphPad Software, USA) was employed for statistical analysis and graph plotting. One-way ANOVA followed by Tukey’s or Dunnett’s multiple comparisons test was used to analyze differences among groups. A *P* value less than 0.05 was considered statistically significant.

## Results

### CircRNA Profile Engaged in NSCLC Development and LNM

The circRNA profile could differentiate NSCLC tissues from adjacent non-cancerous tissues by PCA and heatmap analyses, with 173 upregulated and 282 downregulated circRNAs identified in NSCLC tissues by volcano plot, and their enriched bioprocesses and pathways are presented in **Supplementary Figures 1A–E**. Notably, the circRNA profile could also distinguish NSCLC tissues with LNM from those without LNM, with 183 upregulated and 170 downregulated circRNAs discovered in NSCLC tissues with LNM by volcano plot, and their enriched bioprocesses and pathways are presented at the end of this paper (**Supplementary Figures 1F–J**).

**Figure 1:**
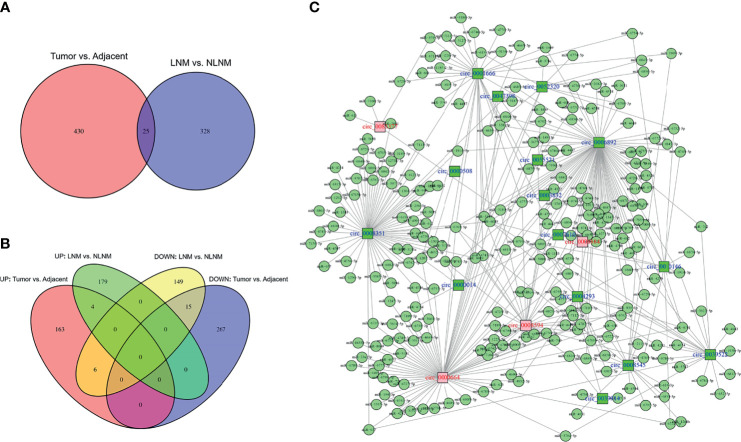
Cross-analyses. Cross-analyses of circRNAs dysregulated in both NSCLC tissues *versus* adjacent non-cancerous tissues and NSCLC tissues with LNM *versus* those without LNM **(A)**. Accordant circRNA analyses by Venn plots **(B)**. Regulatory network of 19 candidate circRNAs **(C)**.

In order to sort candidate circRNAs not only relating to NSCLC development but also linking with NSCLC LNM, cross-analysis was performed, which revealed that 25 circRNAs were dysregulated both in NSCLC tissues compared to adjacent non-cancerous tissues and in NSCLC tissues with LNM compared to those without LNM (**Figure 1A**). Among them, four circRNAs were upregulated in NSCLC tissues compared to adjacent non-cancerous tissues and in NSCLC tissues with LNM compared to those without LNM, while 15 circRNAs were downregulated (**Figure 1B**). Besides, among these 19 (4 + 15) circRNAs, 12 circRNAs exhibited more than five target miRNAs, six circRNAs had 3–5 target miRNAs, and one circRNA only possessed one target miRNA (**Figure 1C**). In addition, detailed information about the 19 candidate circRNAs that were potentially related to both NSCLC development and LNM is presented (**Table 1**).

**Table 1:** Accordant circRNAs in comparison of tumor *vs*. adjacent and LNM *vs.* NLNM.

CircRNA	Tumor *vs.* Adjacent				LNM *vs.* NLNM				Absolute mean Log_2_FC
	Log_2_FC	*P* value	Adjusted *P* value	Trend	Log_2_FC	*P* value	Adjusted *P* value	Trend	
hsa_circ_0008594	1.2359	0.0001	0.0005	UP	1.0963	0.0000	0.0005	UP	1.1661
hsa_circ_0004293	1.5514	0.0001	0.0044	DOWN	0.5970	0.0063	0.0226	DOWN	1.0742
hsa_circ_0003832	1.1446	0.0043	0.0302	DOWN	0.8719	0.0017	0.0086	DOWN	1.0082
hsa_circ_0055521	1.2848	0.0005	0.0098	DOWN	0.6892	0.0006	0.0040	DOWN	0.9870
hsa_circ_0039914	1.2199	0.0010	0.0143	DOWN	0.7023	0.0059	0.0216	DOWN	0.9611
hsa_circ_0007610	1.1651	0.0021	0.0201	DOWN	0.6466	0.0014	0.0074	DOWN	0.9058
hsa_circ_0039522	1.1307	0.0002	0.0063	DOWN	0.6397	0.0040	0.0161	DOWN	0.8852
hsa_circ_0010146	1.0633	0.0033	0.0264	DOWN	0.6823	0.0040	0.0161	DOWN	0.8728
hsa_circ_0052320	0.8919	0.0090	0.0452	DOWN	0.8238	0.0001	0.0010	DOWN	0.8579
hsa_circ_0008351	1.0270	0.0020	0.0199	DOWN	0.6277	0.0006	0.0040	DOWN	0.8274
hsa_circ_0000014	1.0379	0.0012	0.0150	DOWN	0.6167	0.0079	0.0268	DOWN	0.8273
hsa_circ_0008545	1.0378	0.0010	0.0143	DOWN	0.5876	0.0038	0.0154	DOWN	0.8127
hsa_circ_0006892	0.8542	0.0019	0.0195	DOWN	0.7092	0.0000	0.0004	DOWN	0.7817
hsa_circ_0008884	0.9338	0.0094	0.0461	UP	0.5864	0.0069	0.0241	UP	0.7601
hsa_circ_0000508	0.8637	0.0032	0.0261	DOWN	0.6366	0.0003	0.0024	DOWN	0.7501
hsa_circ_0001666	0.8418	0.0051	0.0330	DOWN	0.5883	0.0031	0.0133	DOWN	0.7151
hsa_circ_0047398	0.7966	0.0034	0.0269	DOWN	0.5899	0.0020	0.0095	DOWN	0.6932
hsa_circ_0083377	0.6975	0.0063	0.0369	UP	0.6214	0.0003	0.0025	UP	0.6595
hsa_circ_0000664	0.6970	0.0079	0.0423	UP	0.6134	0.0001	0.0011	UP	0.6552

Accordant circRNAs in comparison of Tumor vs. Adjacent and LNM vs. NLNM were ranked by the absolute mean value of Log_2_FC. circRNA, circular RNA; LNM, lymph node metastasis; NLNM, non-lymph node metastasis; FC, fold change.

### Key Carcinogenic CircRNAs Involved in NSCLC

So as to further explore the function of candidate circRNAs in NSCLC pathogenesis, three top dysregulated circRNAs (circ_0008594, circ_0004293, circ_0003832) involved in NSCLC development and LNM were selected according to the rank of Log_2_FC (**Table 1**), and then functional experiments were performed.

**Figure 2:**
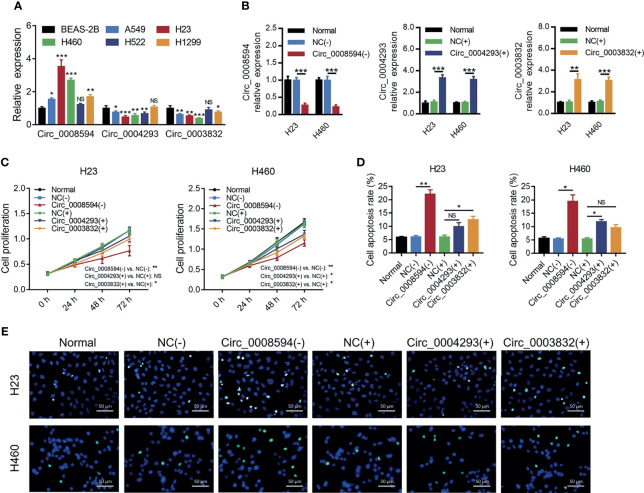
Effects of three candidate circRNAs on NSCLC proliferation and apoptosis. Expression of circ_0008594, circ_0004293, and circ_0003832 by RT-qPCR in NSCLC cell lines and a control cell line **(A)**. Expression of circ_0008594, circ_0004293, and circ_0003832 by RT-qPCR after transfection in H23 and H460 cells **(B)**. Cell proliferation by CCK-8 **(C)** and apoptosis by TUNEL **(D, E)** after transfection in H23 and H460 cells. Each experiment was performed in triplicate. One-way ANOVA followed by Tukey’s or Dunnett’s multiple comparisons test was used for comparison.

Then it was observed that circ_0008594 expression was higher, while circ_0004293 and circ_0003832 expression levels were lower in NSCLC cell lines than in control cells (all *P*<0.05, **Figure 2A**). Interestingly, circ_0008594 knockdown decreased cell proliferation and increased cell apoptosis in both H23 and H460 cells (all *P*<0.05, **Figures 2B–E**). In addition, although overexpression of circ_0004293 and circ_0003832 showed some effect on regulating cell proliferation and apoptosis in H23 and H460 cells, their regulation of these cell functions was weaker than that of circ_0008594 (**Figures 2B–E**). Therefore, circ_0008594 was chosen as a potentially key carcinogenic circRNA engaged in NSCLC development and progression.

### Circ_0008594 Promoted NSCLC Cell Proliferation and Invasion but Affected Stemness Less

**Figure 3:**
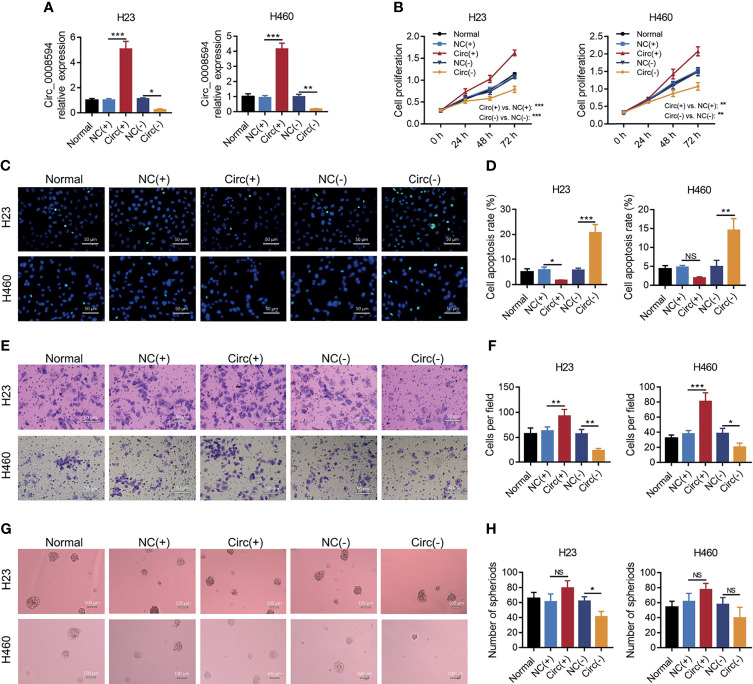
Effects of circ_0008594 on NSCLC proliferation, apoptosis, invasion, and stemness. Comparison of the expression of circ_0008594 by RT-qPCR **(A)**, cell proliferation by CCK-8 **(B)**, cell apoptosis by TUNEL **(C, D)**, cell invasion by transwell **(E, F)**, and sphere formation ability by sphere-formation assay (indicating stemness) **(G, H)** among groups in H23 and H460 cells. Each experiment was performed in triplicate. One-way ANOVA followed by Tukey’s multiple comparisons test was used for comparison.

For the purpose of identifying the role of circ_0008594 in NSCLC pathogenesis, circ_0008594 was modified followed by detection of proliferation, invasion, and stemness in NSCLC cells. It is discovered that circ_0008594 overexpression enhanced H23 and H460 cell proliferation and H23 and H460 cell invasion and reduced H23 cell apoptosis (all *P*<0.05) but did not affect H460 cell apoptosis or H23 and H460 cell sphere formation ability (**Figures 3A–H**). In addition, Circ_0008594 knockdown decreased H23 and H460 cell proliferation, H23 and H460 cell invasion, and H23 cell sphere formation ability and increased H23 and H460 cell apoptosis (all *P*<0.05) but did not influence H460 cell sphere formation ability (**Figures 3A–H**).

### Circ_0008594 Reversely Regulated miR-760 *via* Direct Binding

**Figure 4:**
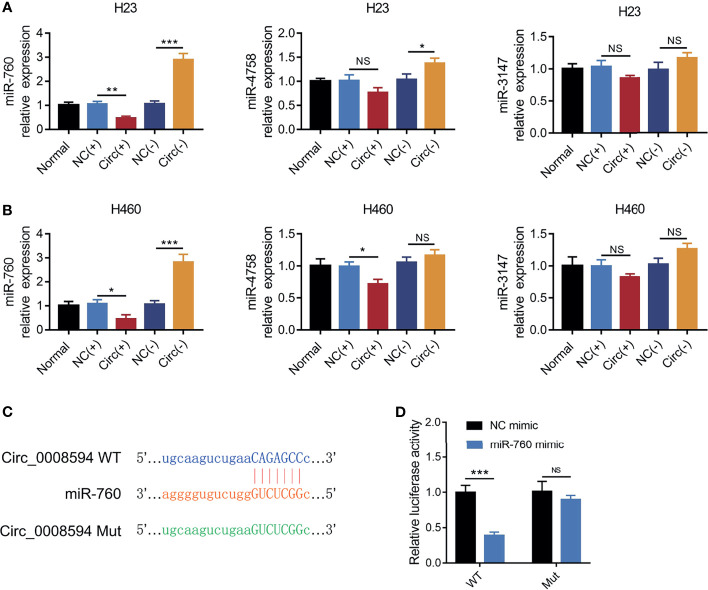
Interaction between circ_0008594 and miR-760. Expressions of miR-760, miR-4758, miR-3147 by RT-qPCR after circ_0008594 modification in H23 and H460 cells **(A, B)**. Luciferase reporter gene assay of the binding between circ_0008594 and miR-760 **(C, D)**. Relative luciferase activity of was normalized to NC mimic group (set as 1). Each experiment was performed in triplicate. One-way ANOVA followed by Tukey’s multiple comparisons test was used for comparison. *P value < 0.05, **P value < 0.01, ***P value < 0.001. ns, not significant.

MiR-760, miR-4758, and miR-3147 are predicted to be the top-ranking targets of circ_0008594, then their expressions were detected after transfection, which exhibited that circ_0008594 overexpression decreased miR-760 expression, while its knockdown increased miR-760 expression in both H23 and H460 cells; besides, miR-4758 and miR-3147 were less affected by circ_0008594 modification in these cells (**Figures 4A, B**). Considering that miR-760 is also reported as a key gene involved in NSCLC etiology (24–26), therefore, the interaction between circ_0008594 and miR-760 was further detected. A luciferase reporter assay revealed that circ_0008594 directly bound to miR-760 (**Figures 4C, D**).

### MiR-760 Compensated for the Effect of Circ_0008594 and Regulated the PI3K/AKT and MEK/ERK Pathways in NSCLC

**Figure 5:**
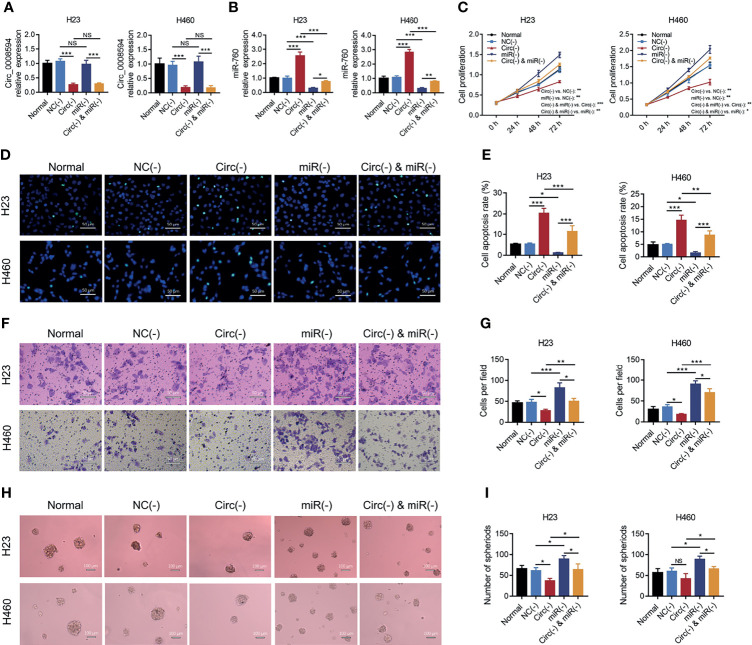
Rescue experiments to detect NSCLC proliferation, apoptosis, invasion, and stemness. Comparison of circ_0008594 expression by RT-qPCR **(A)**, miR-760 expression by RT-qPCR **(B)**, cell proliferation by CCK-8 **(C)**, cell apoptosis by TUNEL **(D, E)**, cell invasion by transwell **(F, G)**, and sphere formation ability by sphere-formation assay (indicating stemness) **(H, I)** among the Normal, NC(−), Circ(−), miR(−), and Circ(−)&miR(−) groups in H23 and H460 cells. Each experiment was performed in triplicate. One-way ANOVA followed by Tukey’s multiple comparisons test was used for comparison. *P value < 0.05, **P value < 0.01, ***P value < 0.001. ns, not significant.

The effect of miR-760 on NSCLC malignant behaviors and its compensative influence on circ_0008594 modification in NSCLC was further explored. It was observed that miR-760 knockdown promoted cell proliferation, invasion, and sphere formation assays but inhibited cell apoptosis in both H23 and H460 cells (all *P*<0.05, **Figures 5A–I**). Notably, miR-760 knockdown also attenuated the effect of circ_0008594 knockdown on NSCLC cell functions (all *P*<0.05, **Figures 5A–I**).

**Figure 6:**
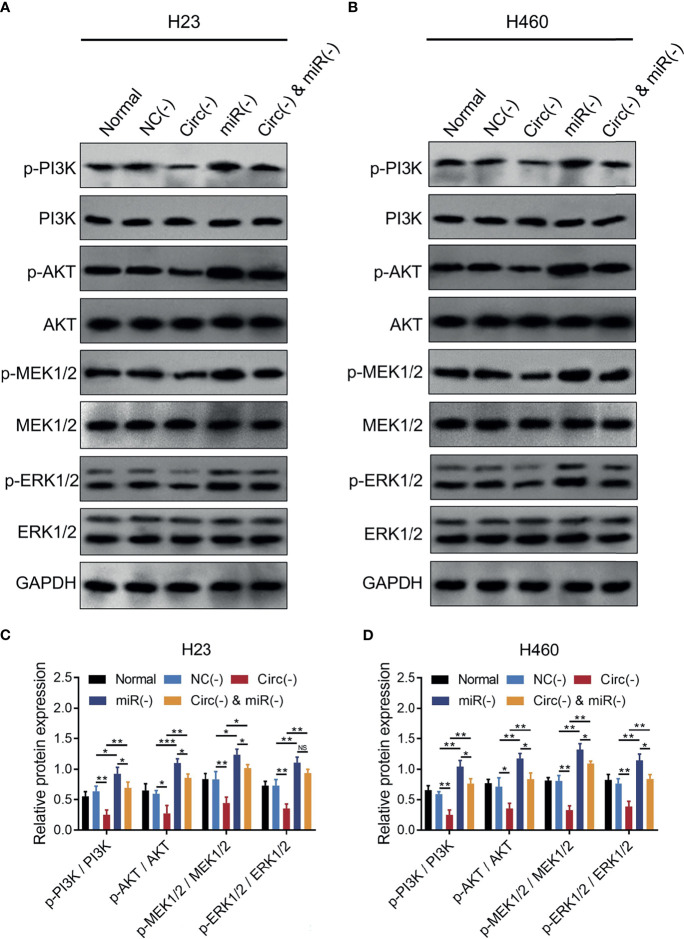
PI3K/AKT and MEK/ERK pathways in NSCLC cells. Comparison of p-PI3K/PI3K, p-AKT/AKT, p-MEK1/2/MEK1/2, and p-ERK1/2/ERK1/2 expression levels by western blot among the Normal, NC(−), Circ(−), miR(−), and Circ(−)&miR(−) groups in H23 **(A, C)** and H460 **(B, D)** cells. Each experiment was performed in triplicate. One-way ANOVA followed by Tukey’s multiple comparisons test was used for comparison. *P value < 0.05, **P value < 0.01, ***P value < 0.001. ns, not significant.

MiR-760 is observed to regulate cancer progression *via* PI3K/AKT and MEK/ERK pathways (25, 27, 28), and the latter pathways are closely implicated in NSCLC progression and stemness (29–32); thus, we further detected the PI3K/AKT and MEK/ERK pathways, which found that circ_0008594 knockdown inactivated the PI3K/AKT and MEK/ERK pathways in both H23 and H460 cells, while miR-760 knockdown showed the opposite effect (all *P*<0.05, **Figures 6A–D**). Furthermore, miR-760 knockdown weakened the effect of circ_0008594 knockdown on regulating the PI3K/AKT and MEK/ERK pathways in H23 and H460 cells (all *P*<0.05).

Meanwhile, ipatasertib (PI3K/AKT inhibitor) greatly weakened the effect of circ_0008594 overexpression on both H23 and H460 cells; trametinib (MEK/ERK inhibitor) also attenuated the effect of circ_0008594 overexpression on both H23 and H460 cells, but the influence was less than ipatasertib (**Supplementary Figures 2A–E**).

### Circ_0008594 Promoted NSCLC Progression and Regulated the miR-760, PI3K/AKT and MEK/ERK Pathways *In Vivo*

**Figure 7:**
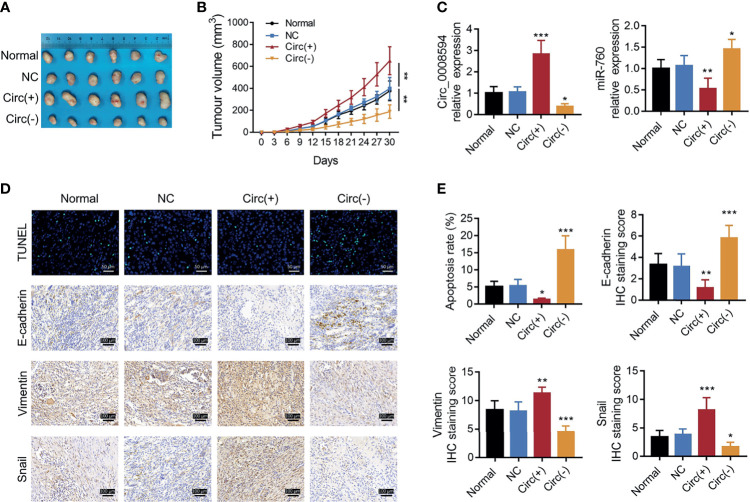
Tumor volume, apoptosis, and EMT markers in NSCLC xenograft models. Images of tumors **(A)**, tumor volumes at different time points **(B)**, expression levels of circ_0008594 and miR-760 by RT-qPCR **(C)**, and tumor apoptosis by TUNEL and EMT markers by IHC **(D, E)** among the Normal, NC, Circ(+), and Circ(−) groups. The IHC staining score was calculated based on intensity and density of stained cells. The intensity was scored as four grades: 0 (negative), 1 (weak), 2 (moderate), and 3 (strong); the density was scored as five grades: 0 (0%), 1 (1–25%), 2 (26–50%), 3 (51–75%), and 4 (76–100%). The final score of IHC assay was a product of the intensity score multiplying the density score. Each group had six mice. One-way ANOVA followed by Dunnett’s multiple comparisons test was used for comparison. *P value < 0.05, **P value < 0.01, ***P value < 0.001.

**Figure 8:**
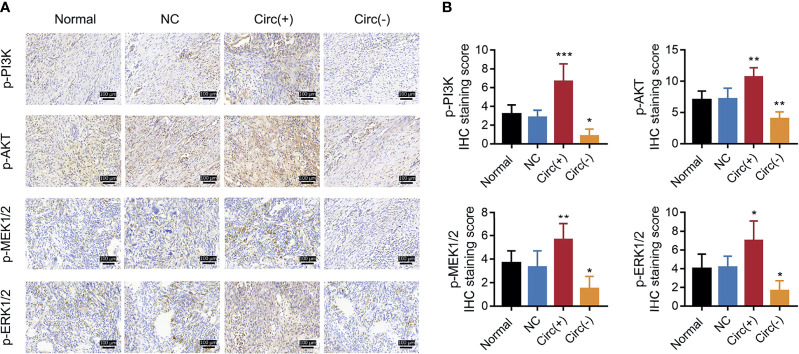
PI3K/AKT and MEK/ERK pathways in NSCLC xenograft models. IHC staining examples **(A)** and IHC scores **(B)** of p-PI3K, p-AKT, p-MEK1/2, and p-ERK1/2 expression levels among the Normal, NC, Circ(+), and Circ(−) groups of NSCLC xenograft models. The IHC staining score was calculated based on intensity and density of stained cells. The intensity was scored as four grades: 0 (negative), 1 (weak), 2 (moderate), and 3 (strong); the density was scored as five grades: 0 (0%), 1 (1–25%), 2 (26–50%), 3 (51–75%), and 4 (76–100%). The final score of IHC assay was a product of the intensity score multiplying the density score. Each group had six mice. One-way ANOVA followed by Dunnett’s multiple comparisons test was used for comparison. *P value < 0.05, **P value < 0.01, ***P value < 0.001.

So as to validate the effect of circ_0008594 on NSCLC progression, *in vivo* experiments were further carried out. We found that circ_0008594 overexpression increased tumor volume, Vimentin expression, and Snail expression, while reduced tumor apoptosis rate, miR-760 expression, and E-cadherin expression in NSCLC xenograft models; moreover, circ_0008594 knockdown exhibited the opposite effect (**Figures 7A–E**). In addition, circ_0008594 overexpression activated the PI3K/AKT and MEK/ERK pathways in NSCLC xenograft models, but its knockdown inactivated these pathways (**Figures 8A, B**).

## Discussion

Our present study observed several new findings regarding circRNAs’ role in NSCLC: (1) *via* microarray analyses, 25 circRNAs were engaged in NSCLC development and LNM; (2) circ_0008594 promoted NSCLC cell proliferation and invasion but affected stemness less; (3) miR-760-mediated PI3K/AKT and MEK/ERK pathways were implicated in the effect of circ_0008594 on NSCLC; and (4) *in vivo* experiments further validated circ_0008594 as a tumor promoter and its interaction with miR-760-mediated PI3K/AKT and MEK/ERK pathways in NSCLC.

Since the introduction of circRNA in cancer research and the progress of genetic detection technology (such as microarray and RNA sequencing), an increasing number of studies have been conducted to investigate the underlying role of the circRNA profile in cancers, including lung cancer (20, 33–36). A previous study identified five upregulated circRNAs and 30 downregulated circRNAs in small-cell-lung cancer (SCLC) tissues compared to adjacent non-cancerous tissues using next-generation sequencing (37). Another study discovered a total of 148 upregulated circRNAs and 23 downregulated circRNAs in NSCLC tissues compared to adjacent non-cancerous tissues by applying a microarray (38). In addition, a bioinformatics analysis involving three GEO datasets (GSE101586, GSE101684, and GSE112214) observed eight dysregulated circRNAs in NSCLC tissues compared to adjacent non-cancerous tissues in these datasets (20). However, these limited previous studies only focused on the dysregulated circRNA profile in NSCLC tissues *versus* adjacent non-cancerous tissues but did not focus on its profile engaged in metastasis; moreover, the sample sizes were relatively low. In our present study, 173 upregulated and 282 downregulated circRNAs were identified in NSCLC tissues compared to adjacent non-cancerous tissues; moreover, 183 upregulated and 170 downregulated circRNAs were discovered in NSCLC tissues with LNM compared to those without LNM. Then, *via* cross-analysis, 19 candidate circRNAs were identified to potentially relate to both NSCLC development and LNM. Among these 19 candidate circRNAs (circ_0008594, circ_0004293, circ_0003832, circ_0055521, circ_0039914, circ_0007610, circ_0039522, circ_0010146, circ_0052320, circ_0008351, circ_0000014, circ_0008545, circ_0006892, circ_0008884, circ_0000508, circ_0001666, circ_0047398, circ_0083377, circ_0000664), only circ_0001666 is previously reported to regulate NSCLC progression *via* miR-330-5p/HMGA2 signaling, while the other 18 circRNAs are not reported in NSCLC (39). Our data provide more evidence for further studies regarding circRNAs in NSCLC etiology and progression.

Circ_0008594 was chosen from three candidate circRNAs (circ_0008594, circ_0004293, circ_0003832) as a potentially key carcinogenic circRNA engaged in NSCLC development and progression *via* functional experiments. Then, the deep underlying mechanism of circ_0008594 was evaluated, and circ_0008594 was found to promote NSCLC cell proliferation and invasion but affected stemness less *in vitro* and enhanced NSCLC tumor growth and EMT *in vivo*. The possible explanations are as follows: circ_0008594 sponged its target miRNAs, such as miR-760, miR-1225, and miR-3180 (as shown in **Figure 1C**), while these miRNAs serve as anti-oncogenes in NSCLC; therefore, circ_0008594 promoted NSCLC growth and invasion (25, 40, 41). Furthermore, we conducted rescue experiments and discovered that the miR-760-mediated PI3K/AKT and MEK/ERK pathways are implicated in the regulatory role of circ_0008594 in NSCLC, which provided novel insight into the mechanism of circ_0008594 in NSCLC pathogenesis.

MiR-760 was previously observed to inhibit the progression of several cancers in multiple ways (25, 42–44). In terms of NSCLC, miR-760 represses NSCLC cell proliferation, the cell cycle and migration by modifying ROS1 (24). In addition, miR-760 retards NSCLC progression *via* the ROS1/Ras/Raf/MEK/ERK pathway (25). Moreover, miR-760 has also been observed to improve sensitivity to TNF-related apoptosis-inducing ligand and radiation therapy in NSCLC (26, 45). Apart from the above papers, other interesting studies also find the role of miR-760 in inhibiting tumor progression and improving drug sensitivity in NSCLC (46, 47). In our present study, we observed that miR-760 knockdown promoted cell proliferation, invasion, and stemness *via* the PI3K/AKT and MEK/ERK pathways *in vitro* and increased tumor growth and EMT *in vivo* in NSCLC, which was in line with previous studies. These results might indicate that miR-760 inactivates several cancer-promoting pathways, such as the ROS1/Ras/Raf/MEK/ERK, PI3K/AKT, cAMP, and Notch1/Hes1/PTEN pathways, to realize its oncogenetic role (25, 27, 28, 48).

In conclusion, our study identifies several valuable circRNAs related to NSCLC development and LNM. Furthermore, as a key functional circRNA, circ_0008594 promotes NSCLC progression by regulating the miR-760-mediated PI3K/AKT and MEK/ERK pathways.

## Strengths and Limitations

Strengths: (1) The current study not only focused on dysregulated circRNA profile in NSCLC tissues *versus* adjacent non-cancerous tissues, but also its profile engaged in metastasis. (2) The key tumor-promoter gene circ_0008594 in NSCLC was sorted by cross-analysis and validated by RT-qPCR and functional experiments. (3) The function and molecule mechanism of circ_0008594 engaged in NSCLC progression was validated by both *in vitro* and *in vivo* experiments.

Limitations: (1) The sample size of clinical parts in our study was relatively small, which precluded a detailed examination of dysregulated circRNA profile in varied histologic subtypes of NSCLC. (2) The correlation of circRNA profile with prognosis of NSCLC was not explored. (3) Although the circ_0008594 was identified as a treatment target in NSCLC according to our findings, whether it would synergize with other treatment could be explored in the future studies.

## Data Availability Statement

The original contributions presented in the study are included in the article/**Supplementary Material**. Further inquiries can be directed to the corresponding author.

## Ethics Statement

The animal experiments were approved by the Animal Care Committee and conducted with the guidelines of the Care and Use of Laboratory Animals.

## Author Contributions

GX conceived and designed the study. QW, CY, PZ, GL, and RZ collected and analyzed the data. HL and LW prepared the figures and tables. QW, CY, PZ, GL, RZ, HL, and LW wrote the manuscript. GX revised the manuscript. All authors contributed to the article and approved the submitted version.

## Funding

This study was supported by the Natural Science Foundation of Heilongjiang Province of China (H2015004).

## Conflict of Interest

The authors declare that the research was conducted in the absence of any commercial or financial relationships that could be construed as a potential conflict of interest.

## Publisher’s Note

All claims expressed in this article are solely those of the authors and do not necessarily represent those of their affiliated organizations, or those of the publisher, the editors and the reviewers. Any product that may be evaluated in this article, or claim that may be made by its manufacturer, is not guaranteed or endorsed by the publisher.

## Supplementary Material

Supplementary Figure 1 Bioinformatics analyses. PCA plot **(A)**, heatmap analysis **(B)**, and volcano plot **(C)** of the circRNA expression profile in NSCLC tumor tissues compared to adjacent noncancerous tissues. GO enrichment analysis **(D)** and KEGG enrichment analysis **(E)** of dysregulated circRNAs between NSCLC tumor tissues and adjacent non-cancerous tissues. PCA plot **(F)**, heatmap analysis **(G)**, and volcano plot **(H)** of the circRNA expression profile in NSCLC tumor tissues with LNM compared to those without LNM. GO enrichment analysis **(I)** and KEGG enrichment analysis **(J)** of dysregulated circRNAs between NSCLC tumor tissues with LNM and those without LNM.

Supplementary Figure 2 Ipatasertib and trametinib treatment. Comparison of cell proliferation by CCK-8 **(A)**, cell apoptosis by TUNEL **(B, C)**, cell invasion by transwell **(D, E)** among the NC(+), Circ(+), Circ(+) and Ipatasertib, Circ(+) and trametinib groups in H23 and H460 cells. Ipatasertib was a PI3K/AKT inhibitor, while trametinib was a MEK/ERK inhibitor. Each experiment was performed in triplicate. One-way ANOVA followed by Tukey’s multiple comparisons test was used for comparison.
