# Supplementary figures and images for: Microarray Identifies a Key Carcinogenic Circular RNA 0008594 That Is Related to Non-Small-Cell Lung Cancer Development and Lymph Node Metastasis and Promotes NSCLC Progression by Regulating the miR-760-Mediated PI3K/AKT and MEK/ERK Pathways
Source: Front Oncol. 2021 Nov 11;11:757541. doi: 10.3389/fonc.2021.757541 (PMC8632265; doi:10.3389/fonc.2021.757541)

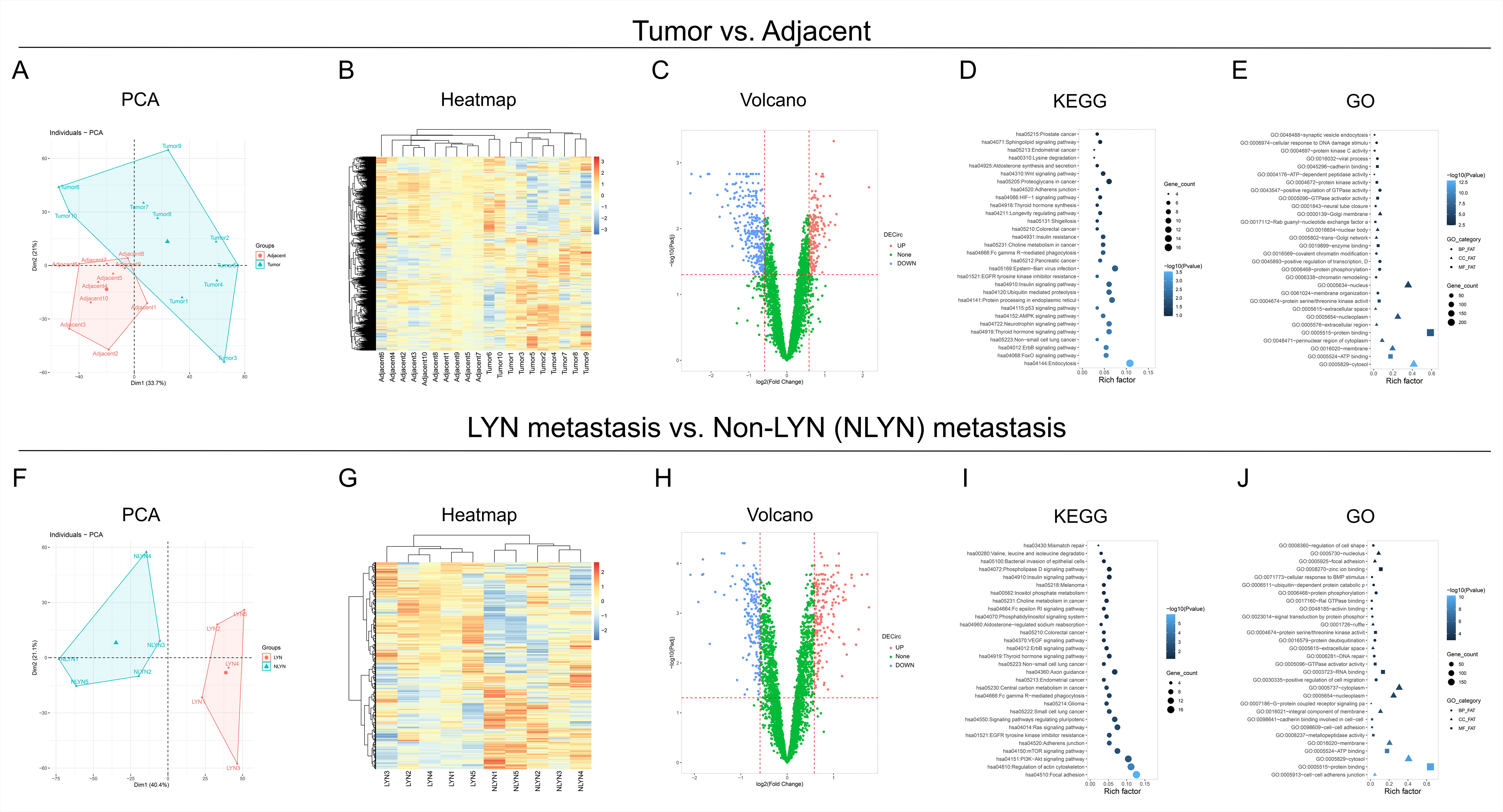

Supplement: Supplementary Figure 1 — Bioinformatics analyses. PCA plot (A), heatmap analysis (B), and volcano plot (C) of the circRNA expression profile in NSCLC tumor tissues compared to adjacent noncancerous tissues. GO enrichment analysis (D) and KEGG enrichment analysis (E) of dysregulated circRNAs between NSCLC tumor tissues and adjacent non-cancerous tissues. PCA plot (F), heatmap analysis (G), and volcano plot (H) of the circRNA expression profile in NSCLC tumor tissues with LNM compared to those without LNM. GO enrichment analysis (I) and KEGG enrichment analysis (J) of dysregulated circRNAs between NSCLC tumor tissues with LNM and those without LNM. [file Image_1.tif]

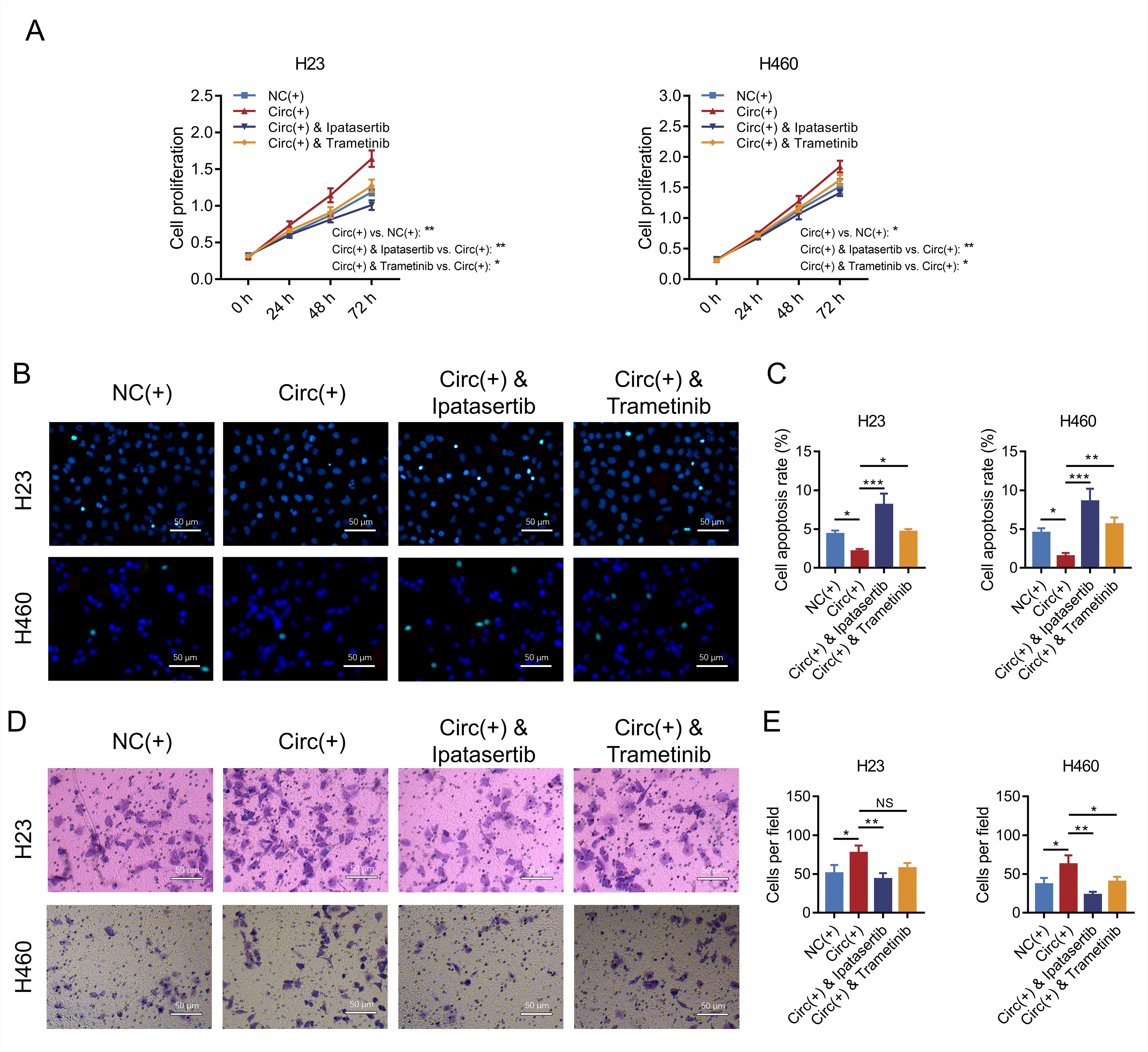

Supplement: Supplementary Figure 2 — Ipatasertib and trametinib treatment. Comparison of cell proliferation by CCK-8 (A), cell apoptosis by TUNEL (B, C), cell invasion by transwell (D, E) among the NC(+), Circ(+), Circ(+) and Ipatasertib, Circ(+) and trametinib groups in H23 and H460 cells. Ipatasertib was a PI3K/AKT inhibitor, while trametinib was a MEK/ERK inhibitor. Each experiment was performed in triplicate. One-way ANOVA followed by Tukey’s multiple comparisons test was used for comparison. [file Image_2.tif]
