# Supplementary material for: Microarray Identifies a Key Carcinogenic Circular RNA 0008594 That Is Related to Non-Small-Cell Lung Cancer Development and Lymph Node Metastasis and Promotes NSCLC Progression by Regulating the miR-760-Mediated PI3K/AKT and MEK/ERK Pathways
Source: Front Oncol. 2021 Nov 11;11:757541. doi: 10.3389/fonc.2021.757541 (PMC8632265; doi:10.3389/fonc.2021.757541)
Supplement: Supplementary file 3 [file Table_1.docx]

**Supplementary Table 1.** Antibodies information of western blot and IHC assays.

| Antibody | Company | Dilution (WB/IHC) |
| --- | --- | --- |
| **Primary Antibody** |  |  |
| Anti-PI3K Antibody | Abcam (USA) | 1:1000/- |
| Anti-p-PI3K Antibody | Abcam (USA) | 1:1000/1:100 |
| Anti-AKT Antibody | Abcam (USA) | 1:1000/- |
| Anti-p-AKT Antibody | Abcam (USA) | 1:1000/1:100 |
| Anti-MEK1/2 Antibody | Cell Signaling Technology (USA) | 1:1000/- |
| Anti-p-MEK1/2 Antibody | Cell Signaling Technology (USA) | 1:2000/1:50 |
| Anti-ERK1/2 Antibody | Cell Signaling Technology (USA) | 1:1000/- |
| Anti-p-ERK1/2 Antibody | Cell Signaling Technology (USA) | 1:2000/1:200 |
| Anti-E-cadherin Antibody | Cell Signaling Technology (USA) | 1:1000/- |
| Anti-Vimentin Antibody | Cell Signaling Technology (USA) | 1:1000/- |
| Anti-Snail Antibody | Cell Signaling Technology (USA) | 1:1000/- |
| Anti-GAPDH Antibody | Affinity (China) | 1:10000/- |
| **Secondary Antibody** |  |  |
| Goat Anti-Rabbit IgG HRP | Affinity (China) | 1:5000/1:200 |
| Goat Anti-mouse IgG HRP | Affinity (China) | 1:5000/1:200 |
