# Supplementary material for: Microarray Identifies a Key Carcinogenic Circular RNA 0008594 That Is Related to Non-Small-Cell Lung Cancer Development and Lymph Node Metastasis and Promotes NSCLC Progression by Regulating the miR-760-Mediated PI3K/AKT and MEK/ERK Pathways
Source: Front Oncol. 2021 Nov 11;11:757541. doi: 10.3389/fonc.2021.757541 (PMC8632265; doi:10.3389/fonc.2021.757541)
Supplement: Supplementary file 4 [file Table_2.docx]

**Supplementary Table 2.** Primers.

| Gene | Forward primer (5'->3') | Reverse primer (5'->3') |
| --- | --- | --- |
| Circ_0008594 | GAGGAAGTGGACCAAGATGCT | TCAAGGTTGTACTCCGCAGAC |
| Circ_0004293 | ACACAGACACAGCAACAGATGA | TGAACGGTAAGCAGCAGTAACT |
| Circ_0003832 | GGTCTCTGTGCAGCCAAAGC | GGTGCCATAGCCTCCAAGAATC |
| miR-760 | ACACTCCAGCTGGGCGGCTCTGGGTCTGTG | TGTCGTGGAGTCGGCAATTC |
| miR-4758 | ACACTCCAGCTGGGGTGAGTGGGAGCCGGT | TGTCGTGGAGTCGGCAATTC |
| miR-3147 | ACACTCCAGCTGGGGGTTGGGCAGUGAGGA | TGTCGTGGAGTCGGCAATTC |
| GAPDH | GAGTCCACTGGCGTCTTCAC | ATCTTGAGGCTGTTGTCATACTTCT |
| U6 | CGCTTCGGCAGCACATATACTA | ATGGAACGCTTCACGAATTTGC |
